# Guest‐Triggered Aggregation‐Induced Emission in Silver Chalcogenolate Cluster Metal–Organic Frameworks

**DOI:** 10.1002/advs.201801304

**Published:** 2018-11-13

**Authors:** Xiao‐Hui Wu, Peng Luo, Zhong Wei, Yuan‐Yuan Li, Ren‐Wu Huang, Xi‐Yan Dong, Kai Li, Shuang‐Quan Zang, Ben Zhong Tang

**Affiliations:** ^1^ College of Chemistry and Molecular Engineering Zhengzhou University Henan 450001 P. R. China; ^2^ Department of Chemistry and Hong Kong Branch of Chinese National Engineering Research Center for Tissue Restoration and Reconstruction The Hong Kong University of Science & Technology Clear Water Bay Kowloon 999077 Hong Kong P. R. China

**Keywords:** aggregation‐induced emission, cluster compounds, crystal transformation, host–guest chemistry, metal–organic frameworks

## Abstract

Utilizing aggregation‐induced emission luminogens (AIEgens) as ligands has proven to be an effective strategy for constructing metal–organic frameworks (MOFs) with intense luminescent properties. However, highly luminescent AIEgen‐based MOFs with adjustable emission properties are rarely achieved because of the rigid conformation of AIEgens in the crystalline state. Here, a dual‐node 3D silver chalcogenolate cluster MOF (**1**) is designed and synthesized, where the AIE ligand shows relatively flexible and rotatable conformations. The conformations of AIE ligands in **1** are switchable by the absorption/desorption of guest molecules. As a result, **1** exhibited not only intense but also guest molecule switched luminescent properties. More importantly, the switching rate is tunable by using different guest molecules. **1** provides a unique visualized prototype to understand the mechanism of guest‐triggered aggregation‐induced emission in MOFs.

Metal–organic frameworks (MOFs) with good luminescent properties have great potential in application areas such as chemical sensors and light‐emitting diodes.^1^ The design and synthesis of new luminescent MOFs has been a hot topic in the past decade. However, the luminous efficiencies of luminescent MOFs are usually limited by the notorious aggregation‐caused quenching phenomenon: most of the luminogens exhibit intense luminescence in dispersed state but weak or even no luminescence in aggregated state and solid state due to the self‐quenching effect caused by π–π stacking of adjacent molecules.^2^ To solve this problem, propeller‐like aggregation‐induced emission luminogens (AIEgens) were introduced to the design of luminescent MOFs in recent years,^3^ which effectively limited the π–π staking of AIEgens in aggregated and solid states.^4^ Meanwhile, the AIEgens were rigidified in the frameworks of MOFs, where the excited state nonradiative transition caused by intramolecular rotations was limited, resulting in intense luminescence.^5^ This process was also known as matrix coordination induced emission.[[qv: 3a]] However, due to the rigid linker conformation, the emission wavelength of AIEgen‐based MOFs is usually quite unitary and guest‐triggered aggregation‐induced emission is rarely achieved, which severely limited their application especially in chemical sensors.

To obtain highly luminescent MOFs with switchable emission properties, the design strategy should be carefully considered. It has been reported that the luminescence properties of AIEgens in crystalline materials are strongly affected by their conformations.^6^ Hence constructing an AIEgen‐based MOFs with tunable conformations of AIEgens might be a feasible option. On one hand, the AIE ligands should have multistage rotors to reduce the impact of coordination on intramolecular rotations.[[qv: 3c]] On the other hand, bigger nodes should be brought into the MOFs, which endow it with higher porosity for conformational variation of AIE ligands.^7^ Based on these two considerations, 1,1,2,2‐tetrakis(4‐(pyridin‐4‐yl)phenyl)‐ethene (tppe) ligands and silver chalcogenolate cluster nodes were combined to design new luminescent MOFs in this work, and a dual‐node 3D silver chalcogenolate cluster MOF (**1**⊃DMAC, DMAC = dimethylacetamide) was successfully synthesized (**Figure** **1**). As a typical AIE ligand, tppe is an ideal option because of its high quantum yield in the solid state.[[qv: 5d,8]] Meanwhile, tppe possesses eight rotors and the inner ones are far away from the nodes, which can effectively avoid steric hindrance induced by coordination. Furthermore, compared with traditional metal nodes, silver chalcogenolate clusters as nodes are more likely to form highly porous structures because of their large size, flexible coordination modes, and variable configurations.^9^

**Figure 1:**
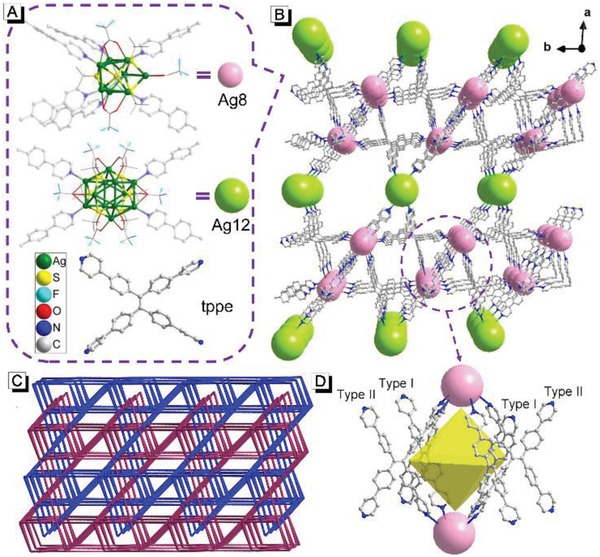
A) Molecular structure of Ag_8_ cluster, Ag_12_ cluster and tppe ligand. B) Single net of **1**⊃DMAC framework viewed along the *c*‐axis, the DMAC molecules are omitted for clarity. C) Two fold interpenetrated topology of **1**⊃DMAC. D) Enlargement of the circled section in (B): an octahedral cage surrounded by four tppe ligands and two Ag_8_ clusters.

Single‐crystal X‐ray analysis reveals that **1**⊃DMAC crystallizes in triclinic space group P‐1, and has a general formula {[Ag_12_(S*^t^*Bu)_6_(CF_3_CO_2_)_6_]_0.5_[Ag_8_(S*^t^*Bu)_4_(CF_3_CO_2_)_4_](tppe)_2_(DMAC)_10_}*_n_* (The number of DMAC molecules that defined from single‐crystal X‐ray diffraction analysis is eight, the other two DMAC molecules are highly disordered, which cannot be well defined). This formula was further confirmed by thermogravimetric analysis (TGA), elemental analysis, and ^1^H‐NMR spectroscopy (See Figure S7 and Figure S8, Supporting Information). As shown in Figure 1, **1**⊃DMAC is built of Ag_8_ cluster (pink balls) and Ag_12_ cluster (green balls) nodes. The Ag_8_ cluster can be considered as a deformed trigonal prism with two silver atoms hanging outside a prismatic edge, which are united by four S*^t^*Bu and four CF_3_CO_2_^−^ auxiliary ligands (Figure S1, Supporting Information). Each Ag_8_ cluster acts as a 6‐connected node, in which each of six silver atoms on the triangular planes is extended by one tppe via Ag—N bonding (Figure 1A). The Ag_12_ cluster node exhibits an irregular sandwich structure (3‐6‐3 conformation) bound by six S*^t^*Bu ligands and six CF_3_CO_2_^−^ ligands (Figure S2, Supporting Information); four of the six silver atoms on the waist are connected by four different tppe ligands, making Ag_12_ a 4‐connected node (Figure 1A). In both Ag_8_ and Ag_12_ cluster nodes, S*^t^*Bu uniformly μ_4_‐bridges four silver centers. Every tppe ligand connects three Ag_8_ clusters and one Ag_12_ cluster, forming a 3D net (Figure 1B). Two such identical 3D nets interpenetrate, resulting in an overall rigid framework that exhibits a 4,4,6‐c 3‐nodal net with the topological point symbol of {4^2^.8^4^}{4^3^.6^2^.8}_4_{4^9^.6^6^}_2_ (Figure 1C). It is noted that every four tppe ligands and two Ag_8_ clusters are combined into an octahedral cage, with two Ag_8_ clusters occupying the vertices and four tppe moieties covering the faces. These four tppe moieties have two different conformations at adjacent positions, which are marked as Type I and Type II respectively (Figure 1D). DMAC molecules distributed in the pores of the framework (Figure S3, Supporting Information). The accessible porosity of **1**⊃DMAC is 40.9% (calculation details can be found in the Supporting Information), upon removal of the guest molecules.

As shown in **Figure** **2**B and Figure S4 in the Supporting Information, the as‐synthesized **1**⊃DMAC exhibited strong blue emission at 470 nm at room temperature (quantum yield 51.1%). When **1**⊃DMAC were exposed in the atmosphere, its fluorescence gradually changed from blue to green and the maximum emission wavelength shifted to 532 nm (quantum yield 59.7%). Interestingly, this fluorescence change was reversible, as it turned back to blue immediately when the atmosphere exposed **1**⊃DMAC was treated with DMAC again (Video 1, Supporting Information). To investigate its fatigue resistance, **1**⊃DMAC was alternatively exposed to the atmosphere and treated with DMAC for 1 min. As shown in Figure 1D, the emission ratio at 470/532 nm remained almost constant after recycling five times, indicating a good fatigue resistance. Meanwhile, the photostability of **1**⊃DMAC was investigated. As shown in Figure S5 in the Supporting Information, the emission intensity at 470 nm of **1**⊃DMAC barely changed after being irradiated with 365 nm light for 3 h, indicating its good photostability.

**Figure 2:**
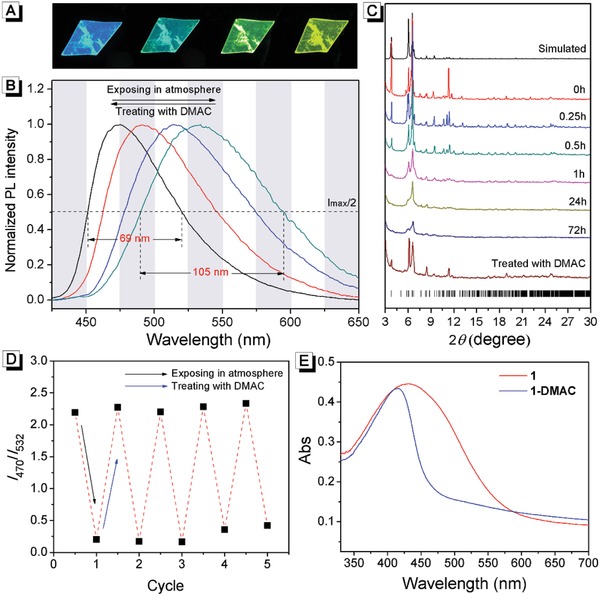
A) Gradual fluorescence changes of the same **1**⊃DMAC crystal under atmospheric exposure. B) Normalized fluorescence spectra of **1**⊃DMAC. C) Time‐dependent PXRD patterns of **1**⊃DMAC exposed in the atmosphere. The bottom purple line is the PXRD pattern of **1** after treatment with DMAC. D) Fatigue resistance of **1**⊃DMAC upon alternative atmospheric exposure and treatment with DMAC. The data were collected from the ratio of fluorescence emission at 470 and 532 nm. E) UV–vis diffuse reflectance spectra of **1**⊃DMAC and **1**.

To understand the mechanism of reversible fluorescence change, the composition of atmosphere‐exposed **1**⊃DMAC was investigated first. As shown in Table S1 in the Supporting Information, the nitrogen content of the atmosphere exposed **1**⊃DMAC was significantly lower than that of the as‐synthesized **1**⊃DMAC. The composition of the atmosphere exposed **1**⊃DMAC agreed well with the general formula ({[Ag_12_(S*^t^*Bu)_6_(CF_3_CO_2_)_6_]_0.5_[Ag_8_(S*^t^*Bu)_4_(CF_3_CO_2_)_4_](tppe)_2_}*_n_*, i.e., the composition of empty framework of **1**. This result suggested that DMAC molecules have escaped from **1**⊃DMAC by exposing in the atmosphere, producing **1**. This result was supported by infrared spectroscopic measurements. As shown in Figure S6 in the Supporting Information compared with **1**⊃DMAC, the peaks belonging to DMAC disappeared in the infrared spectra of **1**. TGA results also confirmed that there was no DMAC in **1** (Figure S7, Supporting Information). More direct evidence for the loss of DMAC from **1**⊃DMAC in the atmosphere was obtained from ^1^H‐NMR spectroscopy. The as‐synthesized **1**⊃DMAC and **1** were dissolved in deuterated dimethylsulfoxide (DMSO‐*d*_6_) and their ^1^H‐NMR spectra were recorded. As shown in Figure S8 in the Supporting Information, for the as‐synthesized **1**⊃DMAC, the proton resonance peaks of tppe and DMAC could be well assigned. Integration of the proton resonance peaks demonstrated that the ratio of tppe and DMAC in **1**⊃DMAC was 2:10, which fitted well with its composition. On the contrary, there was no signal of DMAC in the ^1^H‐NMR spectrum of **1**, while the proton resonance peaks of tppe could still be observed. These results demonstrated that the atmosphere exposed **1**⊃DMAC was actually **1**. Meanwhile, from **1**⊃DMAC to **1**, the morphology of crystal remained unchanged even though its fluorescence emission changed significantly (Figure 2A), which suggested that the framework of the crystal stayed intact. Thus, the only variable for the fluorescence was the composition of **1**⊃DMAC: upon exposure to the atmosphere, DMAC molecules escaped from the pores, resulting in transformation from **1**⊃DMAC to **1** and hence a significant ratiometric fluorescence change.

It is known that the conformations of tetraphenylethene derivatives have great impact on their fluorescence, especially in crystalline materials.[[qv: 3b,6a,d,e,g]] Thus the conformations of tppe in **1**⊃DMAC and **1** might be the determinant factor of their different fluorescence. To understand the relationship between the fluorescence and microstructure, a series of experiments were carried out.

The structure transformation from **1**⊃DMAC to **1** was investigated by powder X‐ray diffraction (PXRD). As shown in Figure 2C, along with the increase of exposure time, **1**⊃DMAC transformed to **1** and the diffraction peaks decreased and even disappeared. This result suggested that **1** has a relatively disorder structure, which might originate from the rotations of phenyl and pyridyl groups since the loss of guest molecules released space around the tppe ligands. When **1** was treated with DMAC for 1 min, the diffraction peaks appeared again, which suggested the recovery of ordered structure of **1**⊃DMAC (Figure 2C, purplish red line). This reversible transformation between **1**⊃DMAC and **1** demonstrated their analogous framework structures. The desorption and desorption isotherms of **1** for N_2_, O_2_, and CO_2_ were measured (Figure S9, Supporting Information). The limited adsorption capacity of **1** for N_2_, O_2_, and CO_2_ also hints that the framework structure of **1** might lose high order and become partially disorder. The loss of guest molecules released space around the tppe ligands in **1**, which endowed it with more flexible twisting angles than that in **1**⊃DMAC. As a result, the tppe ligands in **1** exhibit multiple absorption/emission characteristics which thus gives rise to broader absorption/emission spectra. As shown in Figure 2B, the full width at half maximum of **1** in the emission spectrum was 105 nm, which was much broader than that of **1**⊃DMAC (69 nm). Interestingly, the absorption spectrum of **1** was not only broader than that of **1**⊃DMAC, but also red‐shifted, which indicated its better conjugated structure than **1**⊃DMAC (Figure 2E).^10^

Possible fluorescence decay paths for tppe in the MOFs are proposed in **Figure** **3**A. As shown, the steric hindrance between tppe and DMAC endows **1**⊃DMAC with a more rigid structure than **1**, which prevents the excited state distortion of tppe, resulting in a high‐energy excited state conformation.[[qv: 5b,11]] The energy gap between the excited and ground states of tppe in **1**⊃DMAC (path a) was larger than that in **1** (path b). Thus, the emission wavelength of **1**⊃DMAC was shorter than that of **1**. The proposed decay paths are also supported by fluorescence lifetime measurements. As shown in Figure 3B, the lifetime of **1**⊃DMAC and **1** are 0.59 and 3.28 ns, respectively. The steric hindrance between tppe and DMAC prevents the excited state distortion of tppe in **1**⊃DMAC, resulting in a faster exciton energy release via fluorescence with a shorter lifetime. These results also suggested that the luminescence of the MOFs are not originated from the silver chalcogenolate clusters, whose luminescence lifetimes are normally tens to hundreds of nanoseconds.[[qv: 9h,i,l,m,12]]

**Figure 3:**
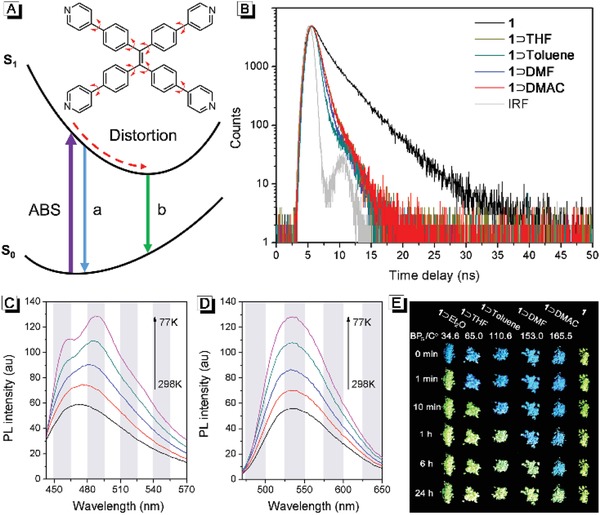
A) Proposed fluorescence decay paths in **1**⊃DMAC (path a) and **1** (path b). B) Fluorescence‐decay profiles of **1**⊃DMAC, **1**⊃THF, **1**⊃Toluene, **1**⊃DMF, and **1**. Fluorescence spectra of C) **1**⊃DMAC and D) **1** at different temperatures. E) Photographic images of fluorescence transformation of **1**, **1**⊃DMAC, **1**⊃DMF, **1**⊃Toluene, **1**⊃THF, and **1**⊃Et_2_O at room temperature. BP_G_ values indicate the boiling points of the guest molecules.

As shown in Figure S3 in the Supporting Information, the determined DMAC molecules were mainly distributed nearby tppe ligands, which indicated that tppe in **1**⊃DMAC were restricted by the incorporated guest molecules, resulting in rigid high‐energy excited state conformations for blue emission. As shown in Figure S10 in the Supporting Information, C‐H···O and C‐H···π weak interactions are found between DMAC and tppe ligands, indicating that there was interaction between solvent molecules and tppe backbone, which may hinder the rotation of the phenyl rings in tppe ligand to trigger the AIE property in the MOFs.

The crystal structure of **1**⊃DMAC suggested that tppe exhibits two conformations (Type I and Type II, as shown in Figure 1D), whose conjugate planes have different but close twist angles (Figure S11, Supporting Information). Thus, theoretically, there should be two different but close fluorescence emissions in **1**⊃DMAC. To get more details on the luminescence characteristics, temperature‐dependent fluorescence spectra of **1**⊃DMAC were recorded. As shown in Figure 3C, there was only one emission peak at 470 nm at 298 K. As the temperature decreased, the emission peak at 470 nm split into two new peaks. One peak was blue‐shifted to 462 nm while the other one was red‐shifted to 488 nm. These two peaks at low temperature demonstrated that different conformations of tppe exhibited different emissions. At the high temperature of 298 K, the thermally activated intramolecular rotations endowed the tppes with similar conformations and hence emission characteristics. In contrast, there was only one emission peak at 532 nm in the fluorescence spectra of **1** at all measured temperatures (Figure 3D). Upon decreasing temperature, the fluorescence intensity increased gradually but the wavelength barely changed, which suggested that there was only one conformation for the tppe in **1**.

To better understand the conformations of tppes in **1**⊃DMAC and **1**, density functional theory calculation was carried out. The optimal conformation of tppe in the free state was obtained and shown in Figure S11 in the Supporting Information (Type III). It was noticed that the angles between the two adjacent C—C single bonds of tetraphenylethene moiety in Type III are 114.5° and 114.4°, respectively, which are highly similar to that in Type I (115.1° and 114.3°) and Type II (115.9° and 114.6°). This result suggested that the rotation of phenyl and pyridyl groups in tppe barely changed their extending directions. That is why the morphologies of **1**⊃DMAC and **1** crystal are analogous (Figure 2A). Meanwhile, the potential energy difference between Type I and Type II was calculated as 1.7 kJ mol^−1^. According to thermodynamics, rotation with an energy barrier of < 2.5 kJ mol^−1^ is free at 298 K.^13^ Thus, at room temperature, tppe ligands in **1**⊃DMAC with Type I and Type II conformations can be transformed into each other and exhibited similar fluorescence emission characteristics. On the contrary, the maximum rotational energy provided by thermal motion was 0.64 kJ mol^−1^ at 77 K, which was lower than the potential energy difference between Type I and Type II. Therefore, the two types of tppe can be clearly distinguished at 77 K (Figure 3C).

According to the above results, the mechanism for reversible transformation between **1**⊃DMAC and **1** was elucidated. In **1**⊃DMAC, there was ordered framework structure constructed by tppe ligands and silver chalcogenolate cluster nodes. Due to the confined effect of DMAC, the tppe ligands were restricted to two different conformations. When DMAC molecules were removed from **1**⊃DMAC, **1** retained its ordered framework structure, and thus the morphology of its crystals remained intact. Meanwhile, the loss of DMAC can release space around the tppe ligands, which allowed the phenyl and pyridyl groups in tppe ligands to rotate to adopt similar conformations. The rotations would make the framework of **1** microscopically slightly deformed, inducing the local disorder structure and the disappearance of some peaks in PXRD patterns. After treatment with DMAC, **1** turned back to **1**⊃DMAC, along with the ordered microstructure again and the recovery of PXRD signals.

To understand the influence of guest molecules on the fluorescence of **1**, three solvents (THF, toluene, and DMF) with different polarity were used for exchange with DMAC from **1**⊃DMAC. First, **1**⊃DMAC was soaked in the selected solvents separately for 1 h. Then the crystals were filtered out to obtain **1**⊃THF, **1**⊃Toluene, and **1**⊃DMF. The ^1^H‐NMR spectra of the exchanged crystals were shown in Figure S12 in the Supporting Information. It was noted that the signals of DMAC disappeared after exchanging with the solvents, while the peaks of exchange solvents could be clearly observed. These results suggested that the DMAC in **1**⊃DMAC had been completely replaced by THF, toluene, and DMF. Infrared spectra also confirmed the formation of **1**⊃THF, **1**⊃Toluene, and **1**⊃DMF. As shown in Figure S13–S15 in the Supporting Information, the peaks of guest molecules could be observed in the corresponding infrared spectra of solvated MOFs. Interestingly, there was no distinct difference observed in the PXRD patterns for **1**⊃DMAC, **1**⊃THF, **1**⊃Toluene, and **1**⊃DMF (Figure S17, Supporting Information), which demonstrated that their frameworks are uniform. Meanwhile, highly analogous emission spectra and fluorescence lifetimes were found in all these MOFs (Figure 3B and Figure S18, Supporting Information). These results demonstrated that the polarity of the guest molecules in **1** have little effect on its fluorescence properties and further confirmed that the steric hindrance between tppe and solvents was the cause of blue fluorescence, which agreed well with the proposed mechanism.

Although their fluorescence characteristics were similar, the switching rates from **1**⊃DMF, **1**⊃THF, and **1**⊃Toluene to **1** were quite different. Inspired by these results, diethyl ether was used to exchange DMAC form **1**⊃DMAC, which have a much lower boiling point and higher volatility than that of the above guest molecules.^14^ As shown in Video 2 in the Supporting Information, the fluorescence of **1**⊃Et_2_O turned from blue to green within 30 s, indicating a fast transformation from **1**⊃Et_2_O to **1**. As shown in Figure 3E, lower boiling point of the guest molecule resulted in a faster switching rate. These results demonstrated that the switching rate can be controllable by changing the guest molecules.

In summary, a dual‐node 3D silver chalcogenolate cluster MOF (**1**) based on AIE ligand was designed and synthesized. **1** exhibited intense and unique guest molecule‐switched fluorescence emission. The easily reversible transformation between the empty framework of **1** and the solvated **1**⊃DMAC led to a ratiometric fluorescence change with good fatigue resistance. In addition, different guest molecules did not vary the fluorescence, but significantly changed the switching rate between **1** and guest‐absorbed **1**. Mechanism study showed that the rotations of phenyl and pyridyl groups in tppe were restricted by the incorporated guest molecules in the pore of **1**, resulting in rigid high‐energy excited state conformations for blue emission; otherwise, the free space for the rotors of tppe in the empty framework of **1** contributed to the other end of the switch– a yellow emission. In all, the reported AIEgen‐based MOFs provide a visualized prototype to understand the mechanism of guest‐triggered aggregation‐induced emission in crystalline materials. Currently, efforts toward developing more luminescent MOFs for sensing and light‐emitting materials are under investigation in our laboratories.

[CCDC 1821839 contains the supplementary crystallographic data for this paper. These data can be obtained free of charge from The Cambridge Crystallographic Data Centre via www.ccdc.cam.ac.uk/data_request/cif.]

## Conflict of Interest

The authors declare no conflict of interest.

## Supporting information

Supplementary

Supplementary

Supplementary
