# Supplementary material for: Guest‐Triggered Aggregation‐Induced Emission in Silver Chalcogenolate Cluster Metal–Organic Frameworks
Source: Adv Sci (Weinh). 2018 Nov 13;6(2):1801304. doi: 10.1002/advs.201801304 (PMC6343058; doi:10.1002/advs.201801304)
Supplement: Supplementary file 1 — Supplementary [file ADVS-6-1801304-s002.pdf]

Copyright WILEY-VCH Verlag GmbH & Co. KGaA, 69469 Weinheim, Germany, 2018.

ADVANCED SCIENCE
Open Access

Supporting Information

for *Adv. Sci.,* DOI: 10.1002/advs.201801304

Guest-Triggered Aggregation-Induced Emission in Silver Chalcogenolate Cluster Metal–Organic Frameworks

Xiao-Hui Wu, Peng Luo, Zhong Wei, Yuan-Yuan Li, Ren-Wu Huang, Xi-Yan Dong, Kai Li, Shuang-Quan Zang,* and Ben Zhong Tang**

Copyright WILEY-VCH Verlag GmbH & Co. KGaA, 69469 Weinheim, Germany, 2016.

Supporting Information

Guest-triggered aggregation-induced emission in silver chalcogenolate cluster metal-organic frameworks

Xiao-Hui Wu, Peng Luo, Zhong Wei, Yuan-Yuan Li, Ren-Wu Huang, Xi-Yan Dong, Kai Li,* Shuang-Quan Zang,* Ben Zhong Tang*

1. Experimental

1.1 Reagents

Unless otherwise noted, all materials used in this work were commercially available and used as received. 1,1,2,2-tetrakis(4-bromophenyl)ethene was purchased from AIEgen Biotech Co., Limited. Palladium(II) acetate, pyridine-4-boronic acid, tricyclohexyl phosphine were purchased from HWRK Chem Co. Beijing, China. Benzenephosphonic acid (PPOA) and tert-butanethiol were purchased from J&K Chemical, Beijing, China. All the other materials were purchased from Sinopharm Chemical Reagent Beijing Co., Beijing, China. Analytical grade solvents include dimethylacetamide (DMAC), dimethyl formamide (DMF), tetrahydrofuran (THF), toluene, diethyl ether (Et_2O) and dichloromethane were used throughout the experiment.

1.2 Apparatus

PXRD data were carried out at room temperature in air using PANalytical-X' Pert PRO diffractometer (Mo-Kα). Thermogravimetry analysis (TGA) of the compounds was conducted on a SHIMADZU TGA-Q50 thermogravimetric analyzer from room temperature to 400 °C at a heating rate of 10 °C/min under N_2 atmosphere. Nuclear magnetic resonance (NMR) data were collected on a Bruker 400 Avance NMR spectrometer operated at 400 MHz at room temperature. The elemental analysis was performed by Thermo Flash EA 1112 Analyzer. Fluorescence spectra, lifetimes and quantum yields were obtained on Edinburgh FLS-980 fluorescence spectrometer. For fluorescence lifetime measurements, a 370 nm-laser was used, which was operated in time-correlated single photon

counting mode (TCSPC) with a resolution time of 100 ns. For fluorescence quantum yields measurements, an integrating sphere was used. Infrared spectra were obtained on PerkinElmer Spectrum Two FT-IR Spectrometer. Ultraviolet-visible diffuse reflectance spectra (UV-DRS) were performed in JASCO-750 UV-vis spectrophotometer, $BaSO_4$ was used as reference. The photos and videos were taken by OLYMPUS BX53 microscope and NIKON D5500 camera.

1.3 Synthesis of 1⊃DMAC.

1,1,2,2-tetrakis(4-(pyridin-4-yl)phenyl)ethane (tppe) was synthesized through a reported procedure.[1] $AgS^{t}Bu$ (0.06 g, 0.203 mmol), $AgCF_3CO_2$ (0.051 g, 0.231mmol) and benzenephosphonic acid (PPOA) (0.06 g,0.378 mmol) were added in 3 mL DMAC in turn. Then tppe (0.005g, 0.0078 mmol) in 3 mL dichloromethane were added. The resultant solution was kept in dark for a couple of days and parallelogram crystals of **1**⊃DMAC was obtained in a yield of 75% (calculated based on tppe), which has a general formula of $[Ag_{12}(S^{t}Bu)_6(CF_3CO_2)_6]_{0.5}[Ag_8(S^{t}Bu)_4(CF_3CO_2)_4](tppe)_2(DMAC)_{10}$.

1.4 TGA analysis

As shown in Figure S6A, the original molecular weight of **1** (**1** with no DMAC) was 4207, while 71% quality remained at 400 °C, which suggested the final molecular weight of **1** was 4207 × 71%=2987. The loss of the weight (4207 × 29% = 1220) was belong to 7 TFA and 7 $S^{t}Bu$ moieties (M = 7 × 113.01 + 7 × 57.11 = 1191). As shown in Figure S6B, the original molecular weight of **1**⊃DMAC (**1** with 10 DMAC) was 5078, while 59% quality remained at 400 °C, which suggested the final molecular weight of **1**⊃DMAC was 5078 × 59%=2996. The loss of the weight (5078 × 41% = 2082) was belong to 10 DMAC, 7 TFA and 7 $S^{t}Bu$ moieties (M = 10 × 87.12 + 7 × 113.01 + 7 × 57.11 = 2062). These two final molecular weights were very close to each other, indicating their same chemical compositions. Both of two TGA results fitted well with the molecular formula of the MOFs, which suggested there were 10 DMAC molecules in **1**⊃DMAC.

1.5 Single crystal X-ray diffraction measurements

Single crystal X-ray diffraction measurement of **1**⊃DMAC sealed in a capillary tube with mother liquor was carried out on a Rigaku XtaLAB Pro diffractometer with Mo-Kα radiation (λ = 0.71073 Å) at 100 K. Data collection and reduction were performed with CrysAlisPro.[2] Multi-scan absorption corrections were applied to the data using CrysAlisPro.[2] The structure were solved with intrinsic phasing methods (*SHELXT-2015*), and refined by full-matrix least squares on F^2 using OLEX2 using the SHELXL-2015 module.[3,4] All non-hydrogen atoms, including the disordered fragments were located in difference-Fourier maps, O atoms, C atoms and N atoms of DMAC molecule were refined isotropically and all other non-hydrogen atoms in the framework were refined

anisotropically. All hydrogen atoms were assigned isotropic displacement coefficients U(H) = 1.2 U or 1.5 U and their coordinates were allowed to ride on their respective atoms. Silver atom Ag_3 and Ag_5 are disordered in the set ratio of 0.80:0.20, the set ratios of Ag_4, Ag_2, Ag_8 are 0.87:0.13, 0.85:0.15 and 0.7:0.3, respectively. The *t*-butyl moiety and trifluoroacetate anions were also found to be disordered. Therefore, the PART instruction was used to divides them into two groups. The least-squares refinement of the structural model was performed under hard geometry restraints and displacement parameter restrains, such as SADI, SIMU, ISOR, DELU, DANG, DFIX and FLAT for the $CF_3CO_2^-$, S^tBu and DMAc molecule. The main solvent peaks were located and refined with the partial occupancies which due to the seriously disorder arising from the thermal motion or dynamic locating of the DMAC in the large solvent accessible space. The remaining unassigned electron densities were removed using SQUEEZE program implemented in PLATON. One Alert B, the missing of FCF reflections below theta (Min), was found in the checkcif file because the low-angle reflections were attenuated by the beam stop. The other three alerts B were ascribed to disordered molecules in the structure, and the short contact between disordered fragments is to be expected. The imposed restraints and constraints in least-squares refinement of the structure were commented in the CIF file. The crystal structure was visualized by Diamond 3.2.[5] Crystal data are summarized in Table S2. The CIF file can be obtained free of charge from the Cambridge Crystallographic Data Centre *via* www.ccdc.cam.ac.uk/data_request/cif (CCDC 1857863 for **1**⊃DMAC).

1.6 Calculation of void space

The void space was calculated from its X-ray structural data by PLATON.[6] The calculation method is as follows: The unit cell contains the atoms from the structural model. Every specific atom is assigned its van der Waals radius, respectively. A list of grid points with a minimum distance of 1.2 Å (from the nearest van der Waals surface) was generated from grid search. Then this list of grid points is applied to produce a new list of grid points, making up the solvent accessible areas. Then the center of gravity and volume of the void are calculated. The volume and center of gravity of individual “voids” are used to calculate the overall solvent accessible volume.

1.7 Density functional theory (DFT) calculation procedure

The DFT calculation were performed with Gaussian 09[7] under M062X functional.[8] The calculations were conducted using 6-31g** basis set for C and N atoms.[9] The single crystal structure of **1**⊃DMAC was chosen as initial guess for ground state optimization.

1.8 Reference

[1] Q. Gong, Z. Hu, B. J. Deibert, T. J. Emge, S. J. Teat, D. Banerjee, B. Mussman, N. D. Rudd, J. Li, *J. Am. Chem. Soc.* **2014**, *136*, 16724-16727.

[2] CrysAlisPro **2012**, Agilent Technologies. Version 1.171.36.31.

[3] G. M. Sheldrick, *Acta Cryst*. *A* **2015**, *71*, 3-8.

[4] O. V. Dolomanov, L. J. Bourhis, R. J. Gildea, J. A. K. Howard, H. Puschmann, *J. Appl. Cryst*. **2009**, *42*, 339-341.

[5] Brandenburg, K. *Diamond*, **2010**.

[6] Spek, A. L. *J. Appl. Crystallogr.* **2003**, *36*, 7-13

[7] M. J. Frisch, G. W. Trucks, H. B. Schlegel, *Gaussian09* **2009**.

[8] Y. Zhao, D. G. Truhlar, *Theor. Chem. Acc.* **2008**, *120*, 215-241.

[9] V. Rassolov, J. A. Pople, M. Ratner, T. L. Windus, *J. Chem. Phys.* **1998**, *109*, 1223-1229.

2. Selected spectra and data referred in the paper

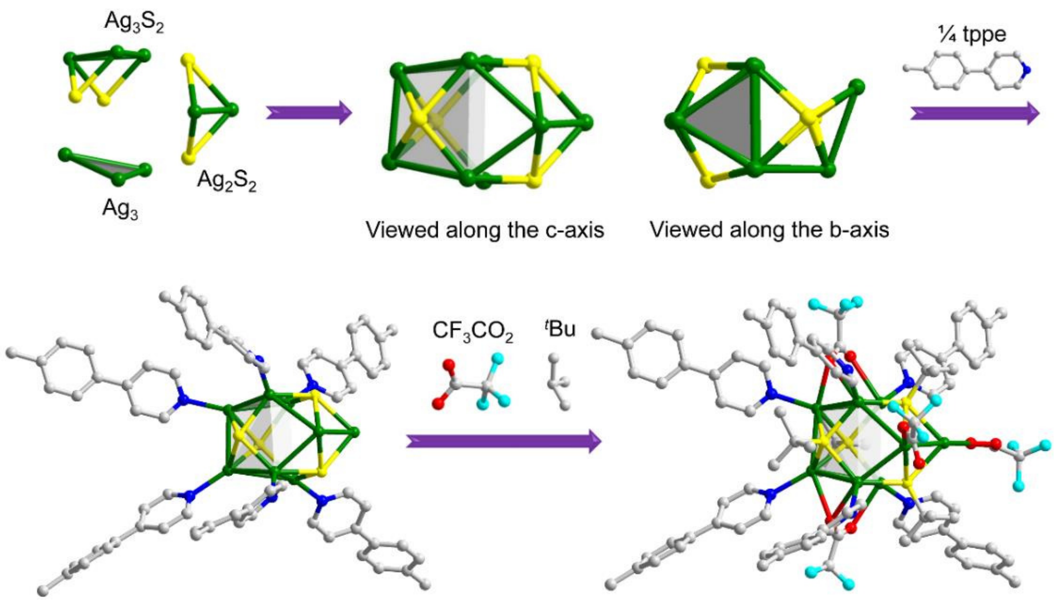

Figure S1. The structure and coordination mode of Ag_8 cluster.

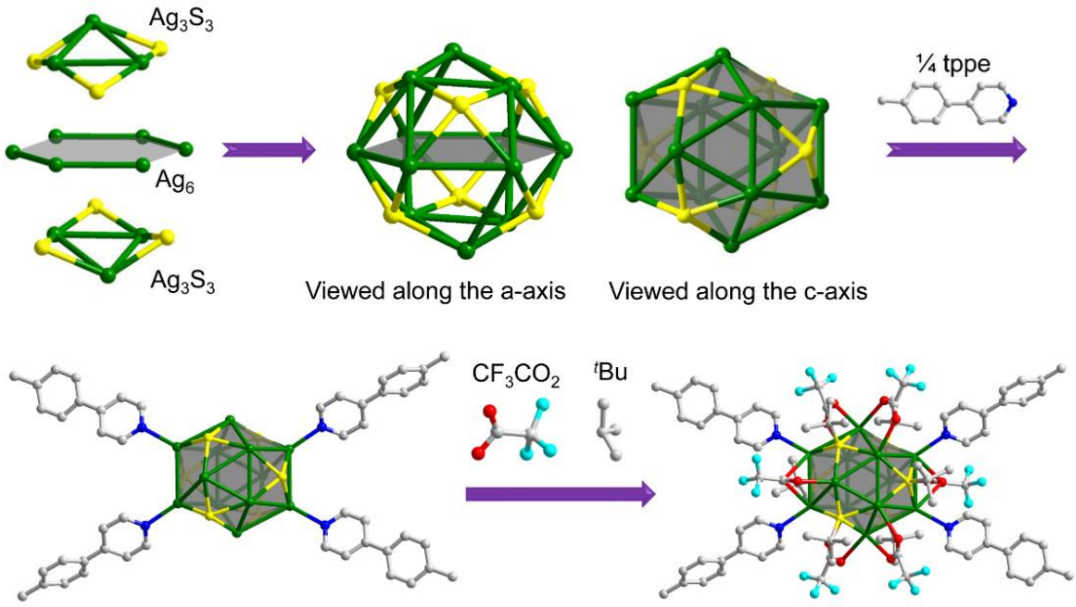

Figure S2. The structure and coordination mode of Ag_{12} cluster.

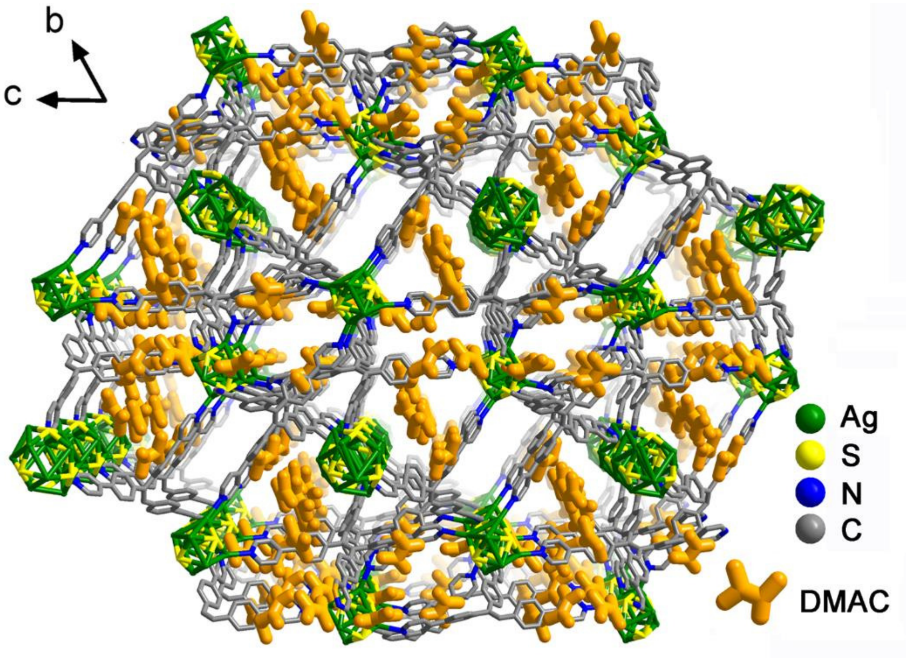

Figure S3. The distribution of DMAC molecules in **1**⊃DMAC.

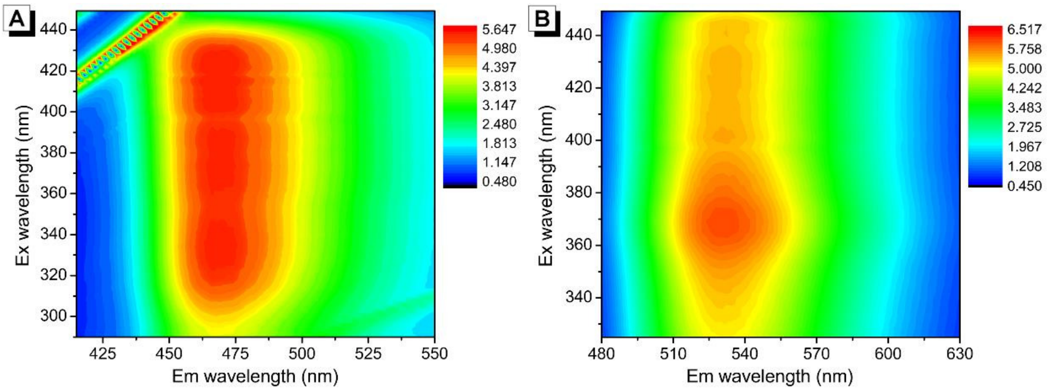

Figure S4. 3D fluorescence spectra of A) **1**⊃DMAC and B) **1**.

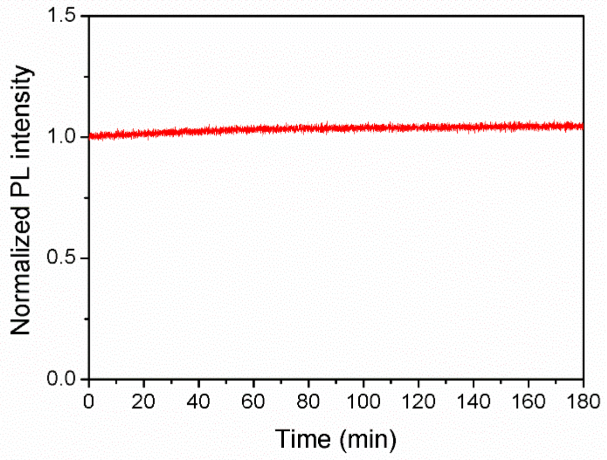

Figure S5. Time dependent fluorescence spectra of **1**⊃DMAC at room temperature. Excitation and emission wavelengths were 365 nm and 470 nm, respectively.

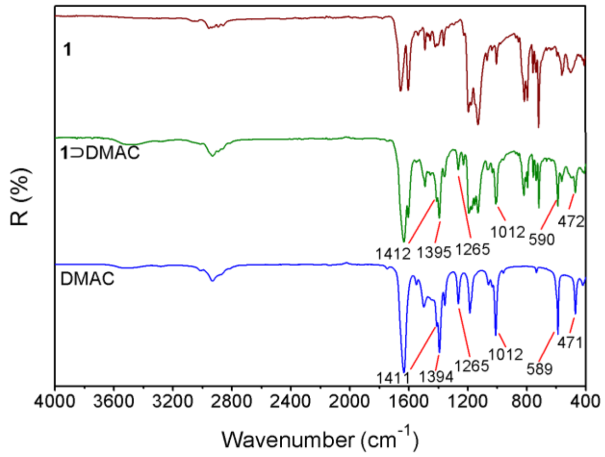

Figure S6. Infrared spectra of **1**, **1**⊃DMAC and pure DMAC.

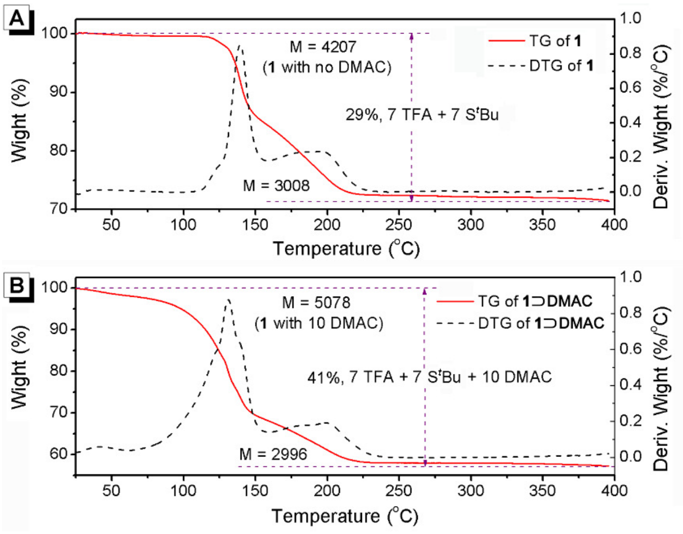

Figure S7. TGA results for as-synthesized A) **1**⊃DMAC and B) **1**.

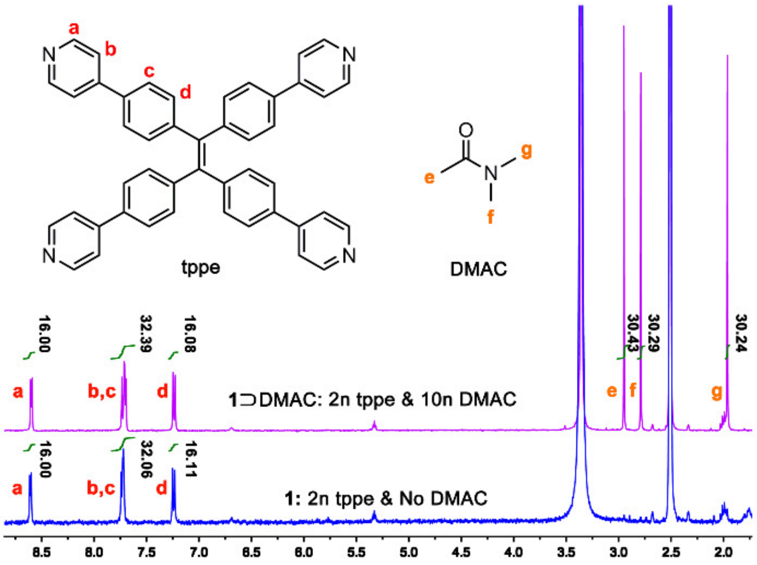

Figure S8. ^{1}H-NMR spectra of as-synthesized **1**⊃DMAC and **1**.

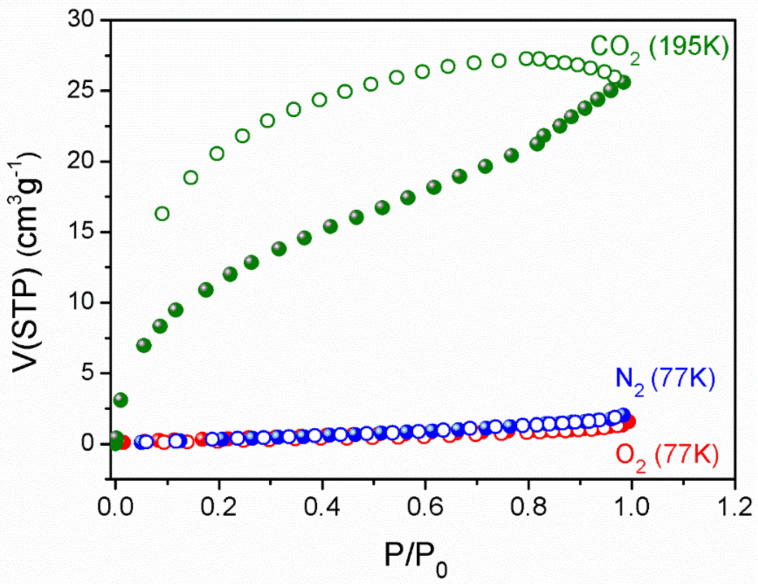

Figure S9. The adsorption (filled symbols) and desorption (empty symbols) of **1** for N_2, O_2 and CO_2.

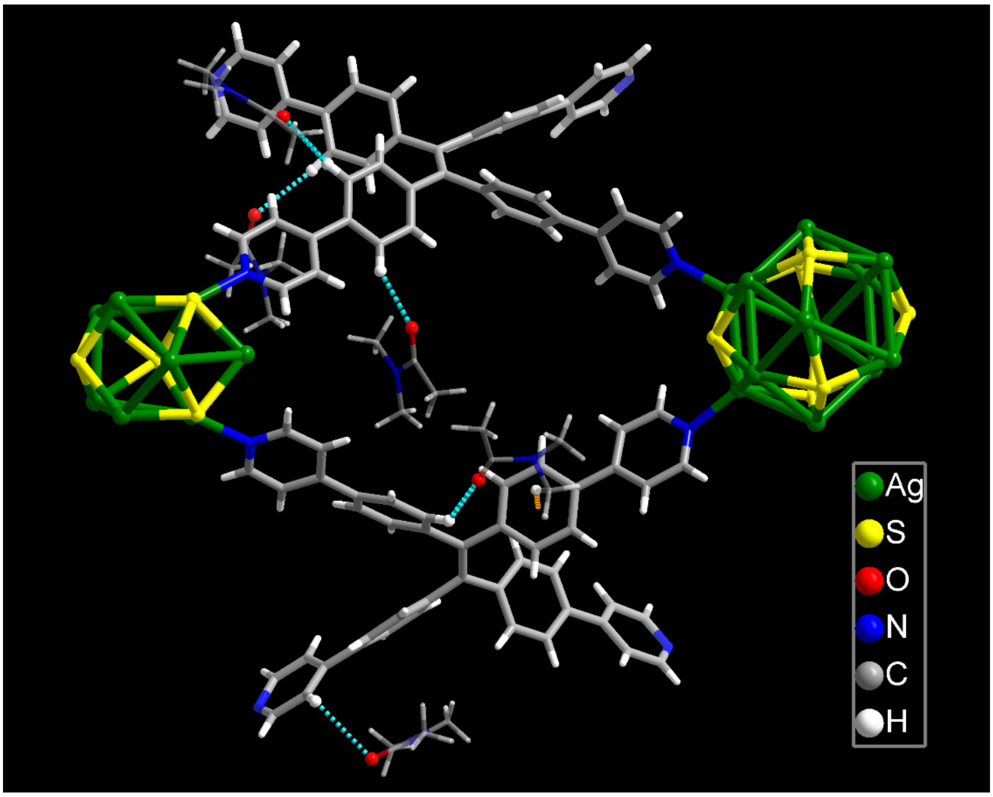

Figure S10. The interactions between DMAC and the tppe backbone. Cyan dash lines are the C-H···O weak interactions. The H···O distances (C-H···O angles) are 2.719Å (118.8°), 2.583Å (132.8°), 2.45Å (151.5°), 2.528Å (162.9°) and 2.512Å (160.8°), respectively. Orange dash line is the C-H···π weak interactions. The H···π distance (C-H···π angle) is 2.757 Å (148.4°).

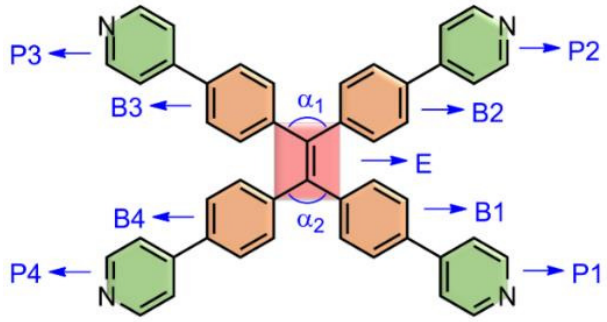

Dihedral angles of the conjugate planes (°)

	Type I	Type II	Type III
α_1	115.1	115.9	114.5
α_2	114.3	114.6	114.4
B1-E	56.2	56.4	49.0
B2-E	48.9	49.5	49.4
B3-E	47.2	43.9	49.0
B4-E	43.2	46.7	49.1
P1-B1	31.8	33.7	35.8
P2-B2	20.1	21.7	37.5
P3-B3	13.2	32.8	35.7
P4-B4	26.4	31.1	35.8

Figure S11. Conformation parameters of tppe ligands in **1**⊃DMAC (Type I and Type II) and the free state (Type III).

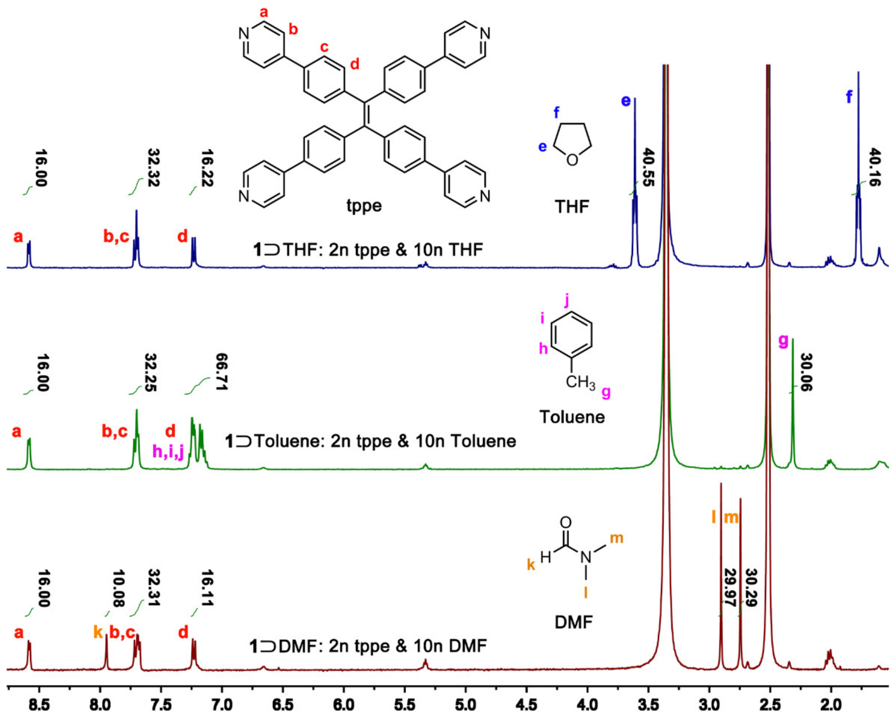

Figure S12. ^{1}H-NMR spectra of **1**⊃THF, **1**⊃Toluene and **1**⊃DMF.

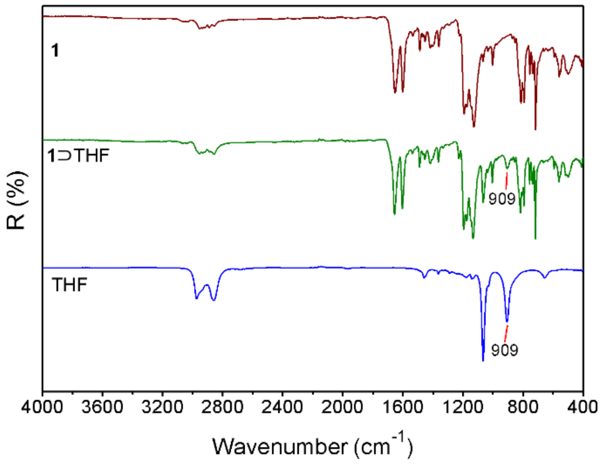

Figure S13. Infrared spectra of **1**, **1**⊃THF and pure THF.

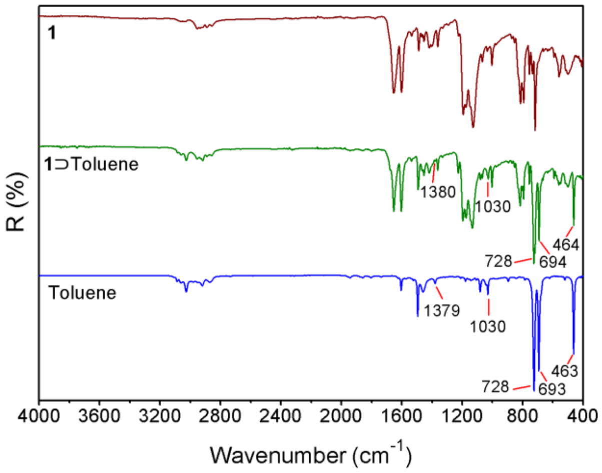

Figure S14. Infrared spectra of **1**, **1**⊃Toluene and pure Toluene.

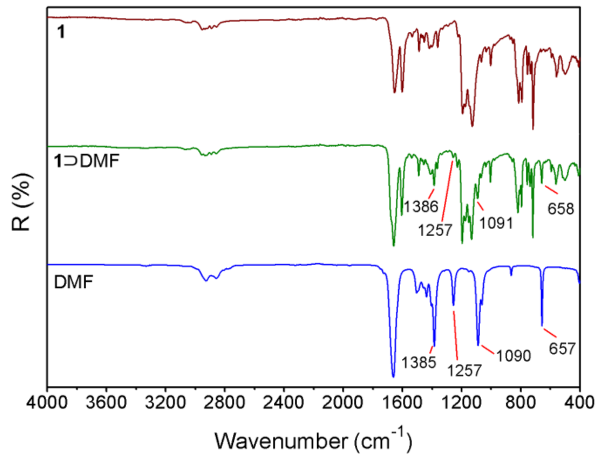

Figure S15. Infrared spectra of **1**, **1**⊃DMF and pure DMF.

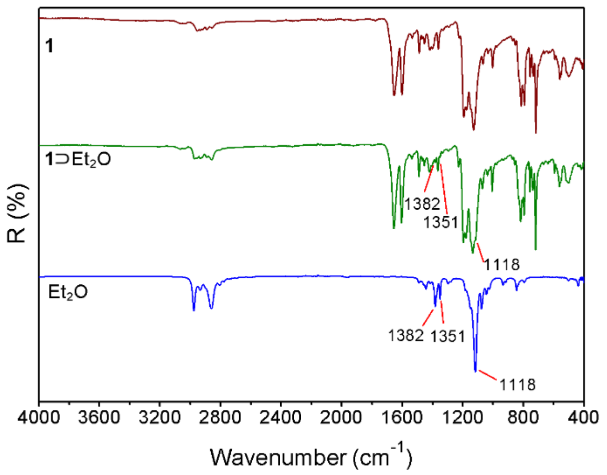

Figure S16. Infrared spectra of **1**, **1**⊃Et_2O and pure Et_2O.

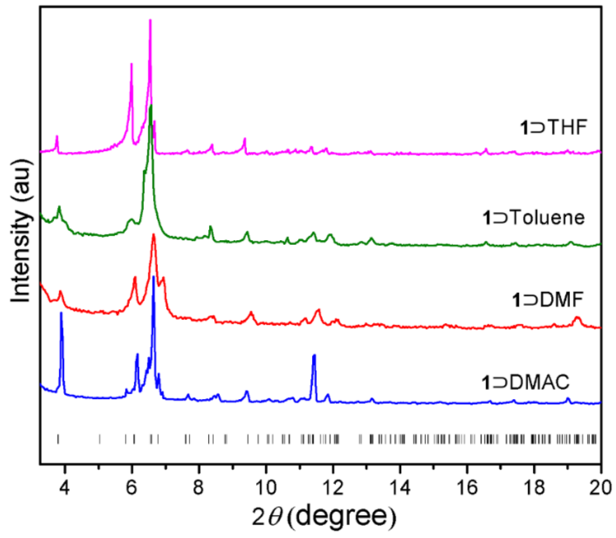

Figure S17. PXRD patterns of **1**⊃THF, **1**⊃Toluene, **1**⊃DMF and **1**⊃DMAC.

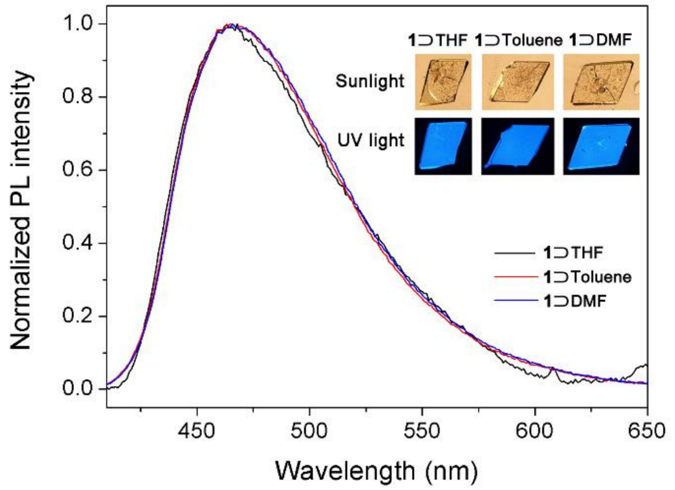

Figure S18. A) Photographic images and normalized fluorescence spectra of **1**⊃THF, **1**⊃Toluene and **1**⊃DMF.

Table S1. Elemental analysis results of **1**⊃DMAC and **1**.

		N (%)	C (%)	H (%)	S (%)
**1**⊃DMAC	Calculated	4.96	41.14	4.31	4.41
	Measured	4.91	41.04	4.54	4.16
**1**	Calculated	2.66	38.25	3.04	5.33
	Measured	2.48	38.10	3.05	5.26

Table S2. Crystallographic data and structure refinement

	**1**⊃DMAC (100K)
CCDC number	1857863
Empirical formula	$C_{152.14}H_{168.44}Ag_{14}F_{21}N_{12.66}O_{18.66}S_7$
Formula weight	4606.51
Temperature / K	100.00(10)
Crystal system	triclinic
Space group	P-1
a /Å	17.5965(2)
b /Å	26.2516(4)
c /Å	26.9520(4)
α /°	60.910(2)
β /°	80.8650(10)
γ /°	89.9000(10)
Volume /Å^3	10700.5(3)
Z	2
ρ_{calc} g/cm^3	1.430
μ /mm^{-1}	1.386
F(000)	4556.0
Crystal size/mm^3	0.18 × 0.15 × 0.12
Radiation	MoKα (λ = 0.71073)
2Θ range for data collection /°	3.862 to 53
Index ranges	$-22 \le h \le 22$, $-32 \le k \le 32$, $-33 \le l \le 30$
Reflections collected	120481
Independent reflections	43452 [R_{int} = 0.0305, R_{sigma} = 0.0420]

Data/restraints/parameters	43452/3788/2231
Goodness-of-fit on F^2	1.084
Final *R* indexes [I>=2*σ* (I)]	R_1 = 0.0760, wR_2 = 0.2188
Final *R* indexes [all data]	R_1 = 0.0994, wR_2 = 0.2301
Largest diff. peak/hole / e Å^{-3}	2.02/-3.18

$R_1 = \sum||F_o|-|F_c||/\sum|F_o|$. $wR_2 = [\sum w(F_o^2 - F_c^2)^2/\sum w(F_o^2)^2]^{1/2}$

3. Caption of videos

Video 1. The transformation from **1** to **1**⊃DMAC.

Video 2. The transformation from **1**⊃Et_2O to **1**.
